# An Iterative Approach for the Parameter Estimation of Shear-Rate and Temperature-Dependent Rheological Models for Polymeric Liquids

**DOI:** 10.3390/polym13234185

**Published:** 2021-11-30

**Authors:** Medeu Amangeldi, Yanwei Wang, Asma Perveen, Dichuan Zhang, Dongming Wei

**Affiliations:** 1 Department of Mathematics, School of Sciences and Humanities, Nazarbayev University, Nur-Sultan 010000, Kazakhstan; medeu.amangeldi@alumni.nu.edu.kz; 2 Department of Chemical & Materials Engineering, School of Engineering and Digital Sciences, Nazarbayev University, Nur-Sultan 010000, Kazakhstan; 3 Laboratory of Computational Materials Science for Energy Applications, Center for Energy and Advanced Materials Science, National Laboratory Astana, Nur-Sultan 010000, Kazakhstan; 4 Department of Mechanical & Aerospace Engineering, School of Engineering and Digital Sciences, Nazarbayev University, Nur-Sultan 010000, Kazakhstan; asma.perveen@nu.edu.kz; 5 Department of Civil & Environmental Engineering, School of Engineering and Digital Sciences, Nazarbayev University, Nur-Sultan 010000, Kazakhstan; dichuan.zhang@nu.edu.kz

**Keywords:** rheology model, polymers, non-Newtonian fluid, time–temperature superposition, curve-fitting, parameter estimation

## Abstract

Numerical flow simulations play an important role in polymer processing. One of the essential prerequisites for accurate and precise flow simulations is to obtain accurate materials functions. In the framework of the generalized Newtonian fluid model, one needs to obtain shear viscosity as a function of the rate-of-shear and temperature—as determined by rheometry—and then fitted to a mathematical model. Often, many subjectively perform the fitting without paying attention to the relative quality of the estimated parameters. This paper proposes a unique iterative algorithm for fitting the rate-of-shear and temperature-dependent viscosity model under the time–temperature superposition (TTS) principle. Proof-of-concept demonstrations are shown using the five-parameter Carreau–Yasuda model and experimental data from small-amplitude oscillatory shear (SAOS) measurements. It is shown that the newly proposed iterative algorithm leads to a more accurate representation of the experimental data compared to the traditional approach. We compare their performance in studies of the steady isothermal flow of a Carreau–Yasuda model fluid in a straight, circular tube. The two sets of parameters, one from the traditional approach and the other from the newly proposed iterative approach, show considerable differences in flow simulation. The percentage difference between the two predictions can be as large as 10% or more. Furthermore, even in cases where prior knowledge of the TTS shifting factors is not available, the newly proposed iterative approach can still yield a good fit to the experimental data, resulting in both the shifting factors and parameters for the non-Newtonian fluid model.

## 1. Introduction

In polymer rheology, the proper non-Newtonian viscosity models are essential for modeling and flow simulations [1]. Experimental measurements of polymeric liquids (solution and melt) are routinely carried out to obtain the necessary data on their rheological properties. Then, various numerical methods were used to find a suitable set of parameters for the rheology model to facilitate efficient flow simulations [2,3,4,5,6,7,8].

In general, there is no universal rule of fitting the rheological data. As stated by Singh et al. (2019) [9], people subjectively choose the fitting approach—thus, leading to non-unique inferences. Moreover, Gallagher et al. (2019) [10] reported the non-identifiability in a family of shear-thinning rheological models. They found a class of parameter sets that fit the data evenly well. Suppose that we choose the rheology model with a large number of parameters. In that case, this issue can arise simply, for instance, from choosing the different sets of the initial guess in the optimization algorithm. One of the traditional optimization algorithms in nonlinear regression is Levenberg–Marquardt (LM) [11,12], but it usually fails with the poor choice of an initial guess. Moreover, Kim et al. [13] advocated the inefficiency of the conventional LM method because of the sometimes unrealistic estimations, which is usually due to the poor choice of an initial guess. Therefore, we need an efficient means of solving the practical issues with fitting the rheological data.

In this paper, we propose the iterative algorithm that helps effectively identify the model parameters. Several research papers presented the algorithms to fit the temperature-dependent rheology model. For instance, Helleloid [14] proposed two numerical methods for obtaining the viscosity–shear rate temperature master curves for polymer fluids. However, the author presented the algorithms with only two experimental viscosity–shear rate data, and the curve fitting was performed by the traditional Newton method. In comparison, here we present our algorithm with more than two viscosity curves and use a more advantageous minimization method. Other authors, Naya et al. [15] developed the method for estimating the optimal shift factors based on the shifting of the derivative functions using the *b*-spline regression and presented reasonably accurate results. However, our algorithm is wholly different and optimizes the shift factors together with the rheology model and is hence a global optimization algorithm.

Part of our algorithm uses the bound-constrained truncated Newton method (TNC) [11] which has both the line search and trust-region frameworks. Unlike the LM method, it searches the optimal values in the trust interval that is set to each of the model parameters. This requirement is vital for realistic parameter estimation. As an example, Venczel et al. [16] used the bound constraints for the parameters in their use of the quasi-Newton numerical method for the Carreau–Yasuda (CY) model. Indeed, setting the constraints is not the sole approach to prevent obtaining unrealistic results. It is also achievable by the direct estimation of the parameters using genetic algorithms from machine learning [17]. As another example, Magnon and Cayeux [18] used the physics-based method to overcome this issue in the context of the Herschel–Bulkley model, and their results were promising at low flow rates. In our case, due to the fast convergence and the possibility of using the constrained approach, we chose the TNC method as an intermediate step in our iterative scheme. In other words, we set lower and upper limit values for the parameters, thus avoiding the unrealistic estimates, for instance, the negative infinite shear-rate viscosity. Simply relying on the theory, we can set the lower limit 0 and upper limit 1 for the parameter *n* in the CY model by shear-thinning [19]. In addition, our global iterative algorithm estimates not only the model parameters but simultaneously the horizontal and vertical shift factors which come from the time–temperature superposition principle (TTS) [20]. Although traditional methods to find the shift factors already exist [21,22,23], our algorithm produces them equally well.

Overall, our approach combines the two-step procedure of finding the model parameters and the shift factors. The quality of the model and its performance in isothermal flows are demonstrated in Section 3.

## 2. Model and Methods

As is well known in polymer rheology, viscosity measurements are taken over a large range of shear rate ($\dot{\gamma }$) values covering several decades of viscosity (η) values. If one attempts to fit the η vs. $\dot{\gamma }$ on a linear–linear scale, the high viscosity values at low shear rates will have a huge influence on the results, whereas the low viscosity values at high shear rates will have almost no effect on the results [24]. The common “trick” in melt rheology, as has been well established, is to fit to the logarithms of the values instead of the values themselves.

Suppose ${\left\{\log_{10}\eta_{i}\right\}}_{i=1}^{m}$ is the set of *m* logged observations of viscosity and ${\left\{\log_{10}{\dot{\gamma }}_{i}\right\}}_{i=1}^{m}$ is the set of *m* logged observations of the shear rate. The five-parameter version of the CY model with the assumption of TTS is given by

(1) $$\begin{array}{c} \eta \left(\dot{\gamma }\right)/a_{T}^{*}=\eta_{\infty }+\left(\eta_{0}-\eta_{\infty }\right){\left[1+{\left(\lambda a_{T}\dot{\gamma }\right)}^{a}\right]}^{\frac{n-1}{a}} \end{array}$$

where $\eta_{\infty }$ is the infinite shear-rate viscosity, $\eta_{0}$ is the zero shear-rate viscosity, $a_{T}$ and $a_{T}*\equiv a_{T}b_{T}$ are the horizontal and vertical shift factors, respectively, and a,n,λ are other constant parameters. We are interested in the minimization of the following sum of the squared error function:

(2) $$\begin{array}{c} S=\sum_{i=1}^{m}{\left[\log_{10}\eta \left(\vec{\theta }\right)-\log_{10}\eta_{i}\right]}^{2} \end{array}$$

where $\vec{\theta }=\left(\theta^{1},\ldots ,\theta^{k}\right)$ is the vector of *k* parameters, particularly the five parameters of the CY model, $\left(\eta_{\infty },\eta_{0},a,n,\lambda \right)$, and $\left\{a_{T}^{*},a_{T}\right\}$ for each temperature and $\eta_{i}$ is the given viscosity at each observation *i*.

### 2.1. Truncated Newton Method

The step-by-step truncated Newton algorithm is described as follows:

*Step 1*: Choose the initial guess ${\vec{\theta }}^{*}$ and compute the function *S*. Set j=0;
*Step 2*: If convergence is satisfied by ${\vec{\theta }}_{j}$, then stop the algorithm;
*Step 3*: Compute the search direction $\vec{p}$ in the matrix Equation:
  $$H\left(S_{j}\right)\vec{p}=-\nabla S_{j}$$
  where $H(S_{j})$ and $\nabla S_{j}$ are the Hessian and the gradient of the function $S({\vec{\theta }}_{j})$, respectively.
*Step 4*: With the $\vec{p}$ obtained in *Step 2*, compute the line search α>0 such that $S\left({\vec{\theta }}_{j}+\alpha \vec{p}\right)<S\left({\vec{\theta }}_{j}\right)$.
*Step 5*: Set ${\vec{\theta }}_{j+1}={\vec{\theta }}_{j+1}+\alpha \vec{p}$, and j=j+1. Return to *Step 2*.

To implement this method, we use the minimization function “TNC” from the Python 3.7 package lmfit of version 1.0.3 [25]. The Python code implementation is available in https://github.com/medeuamangeldi/Combined-iterative-algorithm-with-Hessian-free-optimization-for-nonlinear-data-fitting-/tree/main (accessed on 28 October 2021).

### 2.2. Proposed Global Iterative Algorithm

To demonstrate the potential of the global algorithm, we need to consider two scenarios. Scenario 1 is when we do have the foreknown values of the shift factors, which is often the case in practice, and Scenario 2 is that we do not have the foreknown values of the shift factors. In every fitting, we use the truncated Newton method described in Section 2.1. In addition, the fitted parameters at each iteration are used as the initial guess in the next iteration.

**Scenario** **1.** In this case, suppose we have the known shift factors $a_{T}^{*}$, $a_{T}$, and we use them as the initial guess.

*Step 1*: Construct the master curve with the known shift factors. Fit it with the CY model. Set k=0. If ${\vec{\theta }}_{k}$ is satisfactory for convergence, then terminate the algorithm;
*Step 2*: Fix the estimated parameters ${\vec{\theta }}_{k}$ of (1) except $a_{T}^{*}$, $a_{T}$, and fit the data at each temperature (except the reference) to estimate the shift factors $a_{T}^{*}$ and $a_{T}$ in the Equation (1);
*Step 3*: Construct the master curve with the fitted shift factors from *Step 2*. Fit it with the CY model. Set k=k+1. If ${\vec{\theta }}_{k}$ is satisfactory for convergence, then terminate the algorithm;
*Step 4*: Go to *Step 2*.

**Figure 1:**
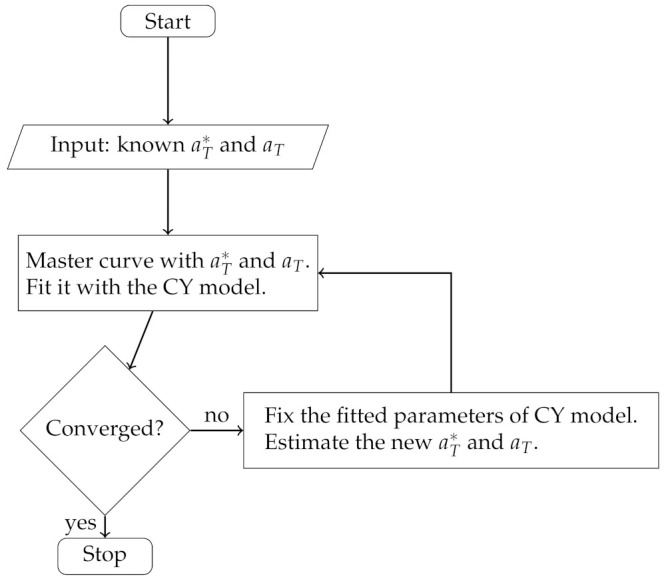
Scenario 1 flowchart.

The algorithm flowchart of Scenario 1 is presented in Figure 1.

**Scenario** **2.** In this case, the description of an iterative approach is as follows:

*Step 1*: Choose the reference temperature. Set $a_{T}^{*}=a_{T}=1$ in Equation (1). Fit the viscosity vs. shear rate data at reference temperature with (1). Set k=0;
*Step 2*: Fix the estimated parameters ${\vec{\theta }}_{k}$ of (1) except $a_{T}^{*}$, $a_{T}$, and fit the data at each temperature (except the reference) to estimate the shift factors $a_{T}^{*}$ and $a_{T}$ in the Equation (1);
*Step 3*: Construct the master curve with the fitted shift factors from *Step 2*. Fit it with the CY model. Set k=k+1. If ${\vec{\theta }}_{k}$ is satisfactory for convergence, then terminate the algorithm;
*Step 4*: Go to *Step 2*.

**Figure 2:**
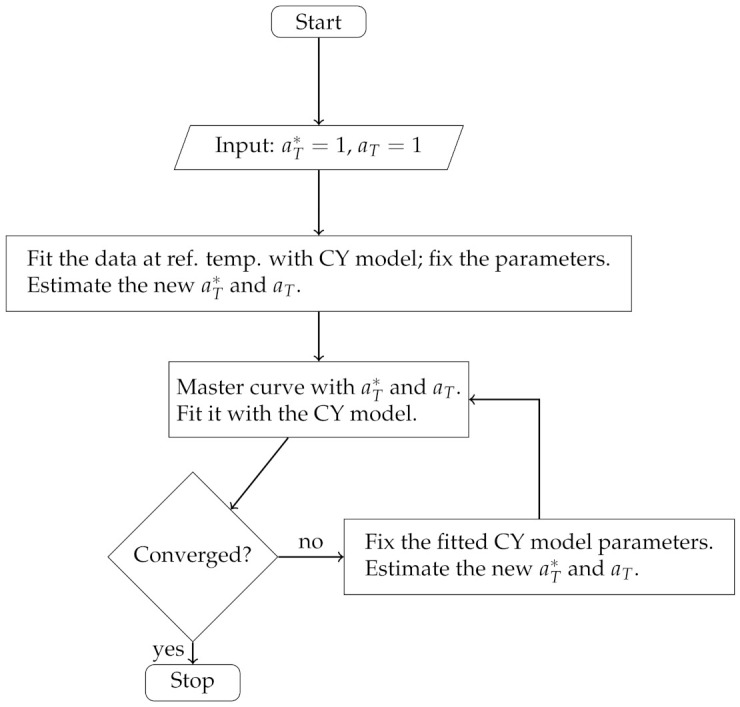
Scenario 1 flowchart.

The algorithm flowchart of Scenario 2 is presented in Figure 2.

### 2.3. Goodness of Fit

In order to measure how well the collected data are replicated by the CY model, we used 4 different estimators—Akaike Information Criterion (AIC) [26], Bayesian Information Criterion (BIC) [27], adjusted R-squared ($R_{\mathrm{adj}}^{2}$) [28] and root mean squared error (RMSE) [29].

The information criteria—AIC—helps identify the relative quality of every fitted CY model. In other words, given the set of models, the most preferred is the one with the lowest AIC value. The equation of AIC is described as

(3) $$\begin{array}{c} \mathrm{AIC}=2k-2\ln\hat{L} \end{array}$$

where *k* is the number of estimated parameters in the CY model and $\hat{L}$ is the maximum likelihood function of the model. The idea of BIC is similar to AIC but with the larger penalty term. It is defined as

(4) $$\begin{array}{c} \mathrm{BIC}=k\ln n-2\ln\hat{L} \end{array}$$

where *n* is the number of data points.

Another measure of model quality is $R_{\mathrm{adj}}^{2}$, the so-called “coefficient of determination”. It is formally defined as

(5) $$\begin{array}{c} R_{\mathrm{adj}}^{2}=1-\left[1-\frac{\sum_{i}{\left(\log_{10}y_{i}-\log_{10}{\hat{y}}_{i}\right)}^{2}}{\sum_{i}{\left(\log_{10}y_{i}-\bar{\log_{10}y}\right)}^{2}}\right]\frac{n-1}{n-p-1} \end{array}$$

where $\left\{y_{i}\right\}$ is a set of observed data, $\left\{{\hat{y}}_{i}\right\}$ is a set of predicted data, $\bar{\log_{10}y}$ is the mean of logged observations, *n* is a sample size and *p* is a number of parameters in the model. We use this estimator as a stopping criteria in our global iterative algorithm by the percent change defined as

(6) $$\begin{array}{c} R_{\mathrm{adj},\%}^{2}=\frac{{\left(R_{\mathrm{adj}}^{2}\right)}_{\mathrm{new}}-{\left(R_{\mathrm{adj}}^{2}\right)}_{\mathrm{old}}}{{\left(R_{\mathrm{adj}}^{2}\right)}_{\mathrm{old}}} \end{array}$$

The last estimator—RMSE—provides the model’s predictive quality by aggregating the differences between the actual and predicted values. It is defined as

(7) $$\begin{array}{c} \mathrm{RMSE}=\sqrt{\frac{\sum_{i=1}^{n}{\left(\log_{10}y_{i}-\log_{10}{\hat{y}}_{i}\right)}^{2}}{n}} \end{array}$$

where $\left\{y_{i}\right\}$ is a set of actual values, $\left\{{\hat{y}}_{i}\right\}$ a set of predicted values and *n* is a sample size. The description of RMSE in the logarithmic space is the same as taking the root of the mean of the squared ratio between the actual and predicted values.

### 2.4. Experimental Data

In this paper, we used the viscosity and shear rate data of polystyrene melt, which was obtained by small amplitude oscillatory shear (SAOS) measurements by Huang et al. [30]. The values were approximated with complex viscosity vs. angular frequency by the Cox–Merz rule [1,31]. In addition to the experimental data, the shift factors were also obtained using the TRIOS software package from TA Instruments.

## 3. Results and Discussion

### 3.1. Nonlinear Regression: Traditional Approach vs. Scenario 1

**Figure 3:**
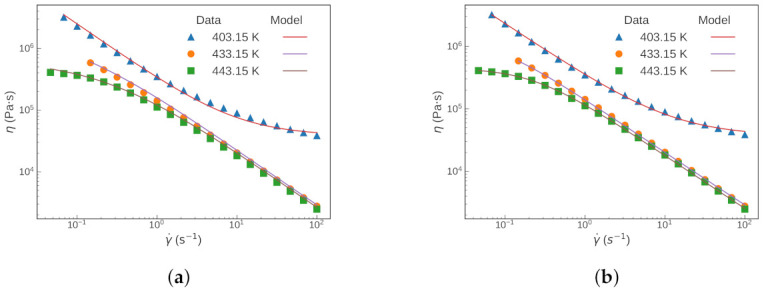
Fitted Carreau—Yasuda model at different temperatures with: (**a**) the traditional approach; and (**b**) Scenario 1.

**Figure 4:**
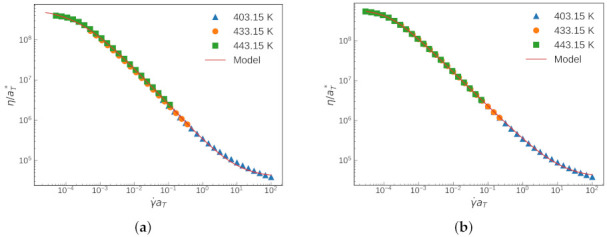
Master curve and the corresponding Carreau—Yasuda fit obtained by: (**a**) traditional approach; and (**b**) Scenario 1.

Usually, obtaining the shift factors is performed independently from the parameter estimation of the rheology model. We demonstrated the results of this traditional approach by fitting the master curve with the existing shift factors. Then, the comparison was made with the results from Scenario 1. At first glance, both approaches gave rather good fits to the experimental data at each of the three temperatures (see Figure 3 and Figure 4).

**Figure 5:**
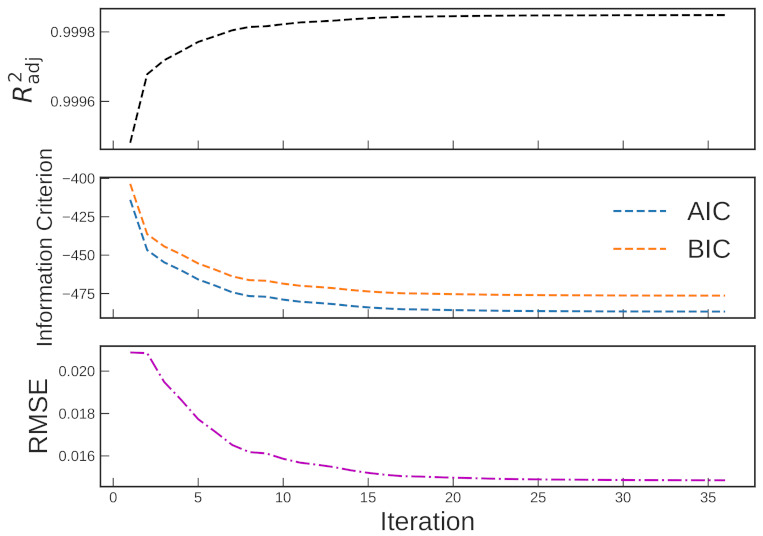
Evolution of various goodness-of-fit values (Scenario 1).

**Figure 6:**
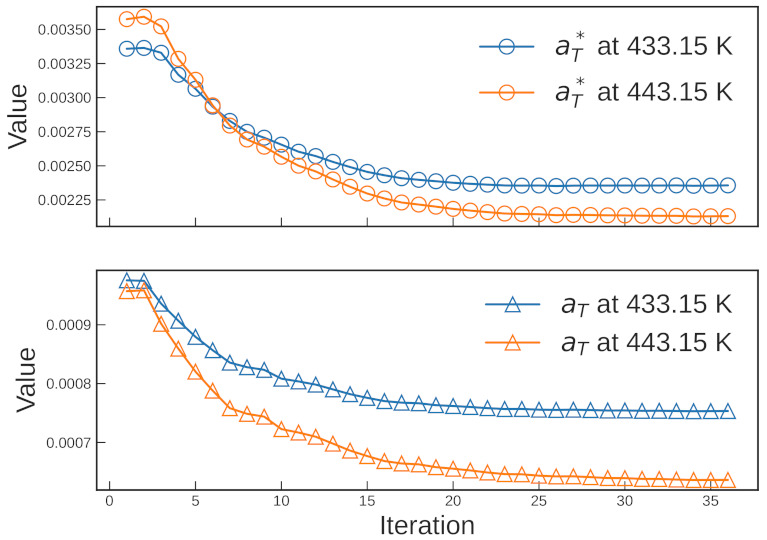
Evolution of the TTS shift factors (Scenario 1).

The evolution of the goodness-of-fit measures of Scenario 1 is shown in Figure 5. They all show monotonic behavior towards a better fitting as the iteration proceeds. The evolution of the TTS shifting factors is shown in Figure 6. As the iteration proceeds, convergence behavior is found for all parameters. Table 1 summarizes the evolution of the estimated parameters of the CY model and shift factors with each iteration (Scenario 1). The values shown for item #1 are referred to as the traditional approach.

As summarized in Table 2, the model by the traditional method has a relatively lower quality of fitting than that in the Scenario 1 by looking at the AIC, BIC and the $R_{\mathrm{adj}}^{2}$ values. Moreover, the RMSE is approximately twice as large as the RMSE of Scenario 1, demonstrating the superior performance of the proposed global iterative algorithm. As a further comparison, Table 2 also presents the performance of the more conventionally used LM method. Judging from all four measures of the goodness-of-fit, the traditional approach using the LM method shows an even poorer performance.

**Figure 7:**
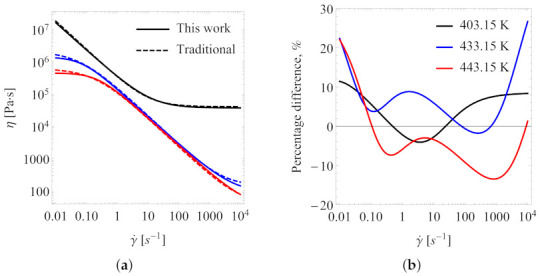
(**a**) Viscosity vs. shear rate at three different temperatures: traditional method (- - - -) and Scenario 1 (—); and (**b**) percentage difference between the two model predictions.

Figure 7 presents a direct comparison between the two sets of the best fitting parameters, one from the traditional approach (as can be seen in #1 in Table 1) and the other from the herein proposed iterative approach, referred to as “Scenario 1” (as can be seen in #37 in Table 1) for the three temperature values of the measurements. Figure 7a shows the shear viscosity vs. shear rate, and Figure 7b shows the percent difference, which is defined as

(8) $$PD=\frac{\eta_{0}-\eta_{1}}{(\eta_{0}+\eta_{1})/2}\times 100\%$$

where $\eta_{0}=\eta_{0}\left(\dot{\gamma }\right)$ and $\eta_{1}=\eta_{1}\left(\dot{\gamma }\right)$ are the predicted shear viscosity from the traditional approach (subscript “0”) and from the method of Scenario 1 (subscript “1”), respectively. From the range of the shear rate of the actual measurement (0.1 to $10^{2}\;s^{-1}$), the percentage difference between the two model predictions is within 10%. However, at a shear rate lower than $10^{-2}\;s^{-1}$ and larger than $10^{3}\;s^{-1}$, the difference between the two model predictions can exceed 10%.

### 3.2. Isothermal Flow in a Tube

In this subsection, we compared the two sets of parameters, one from the traditional approach and the other from the newly proposed iterative approach, in the flow of a CY fluid in a circular tube problem. Tube flow is encountered in several polymer processes [32]. For simplicity, it is assumed that the flow is isothermal (no viscous dissipation), steady, fully developed, with no entrance effects and axis-symmetric. The axial momentum equation is reduced to [1,32]:

(9) $$0=-\frac{dP}{dz}-\frac{1}{r}\frac{d}{dr}\left(r\tau_{rz}\right)$$

where dP/dz=−ΔP/L is the pressure gradient, and $\Delta P=(P_{0}-P_{L})/L$ is the pressure difference over the tube length *L*. The above equation may be integrated to give the rz-component of the viscous shear stress:

(10) $$\tau_{rz}=\frac{1}{2}\frac{\Delta P}{L}r$$

In the framework of the generalized Newtonian fluid (GNF) model, we have:

(11) $$\tau_{rz}=\eta \left(\dot{\gamma }\right)\dot{\gamma }$$

where the shear rate $\dot{\gamma }$ is given by

(12) $$\dot{\gamma }=-\frac{dv_{z}\left(r\right)}{dr}$$

and is a positive quantity. For the CY fluid model, $\eta (\dot{\gamma })$ is given by Equation (1). To sum up, the problem at hand to solve the following first-order nonlinear ODE si:

(13) $$\frac{1}{2}\frac{\Delta P}{L}r=a_{T}^{*}\left\{\eta_{\infty }+\left(\eta_{0}-\eta_{\infty }\right){\left[1+{\left(\lambda a_{T}|\frac{dv_{z}}{dr}|\right)}^{a}\right]}^{\frac{n-1}{a}}\right\}\left(-\frac{dv_{z}}{dr}\right)$$

subject to the non-slip boundary condition of $v_{z}\left(r=R\right)=0$. In this work, we numerically solved the governing ODE, Equation (13), and the accuracy of our numerical solutions were validated by a recently developed analytical solution to this problem [33]. To obtain the numerical solutions, we used Wolfram Mathematica (“12.1.1 for Microsoft Windows (64-bit) (19 June 2020)”). The corresponding Wolfram Mathematica notebook is available in the Supporting Material of this article.

**Figure 8:**
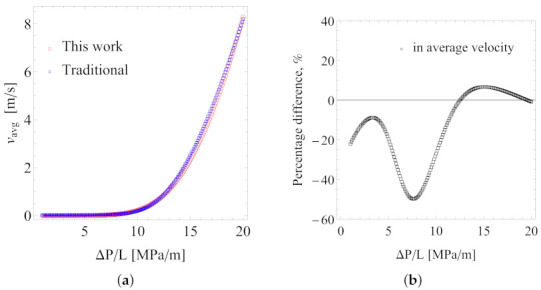
Average flow velocity (**a**) and the percentage difference (**b**) on the predicted average flow velocity. Tube radius *R* = 0.05 rm.

**Figure 9:**
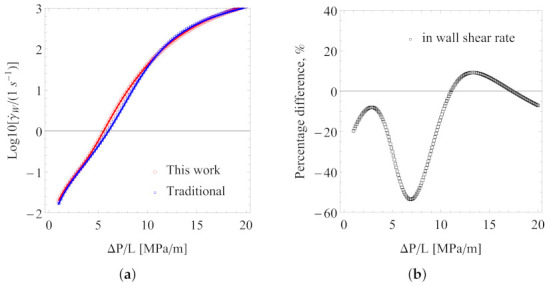
Wall shear rate (**a**) and the percentage difference (**b**) on the predicted wall shear rate. Tube radius *R* = 0.05 rm.

Consider the viscosity data for the intermediate temperature value, T=433.15K, as an example. For a circular tube of radius R=0.05rm, Figure 8a presents the predicted average flow velocity ($v_{\mathrm{avg}}$) as a function of the pressure gradient (ΔP/L) for the two sets of the best fitting parameters. At first glance, they give very similar results. However, upon closer inspection, when the pressure gradient is low, the percentage difference between the predicted average velocities may exceed 40%, as shown in Figure 8b. Here, the percentage difference is defined as

(14) $$\mathrm{PD}=\frac{v_{\mathrm{avg},0}-v_{\mathrm{avg},1}}{(v_{\mathrm{avg},0}+v_{\mathrm{avg},1})/2}\times 100\%$$

where $v_{\mathrm{avg},0}$ and $v_{\mathrm{avg},1}$ are the predicted average flow velocity using viscosity parameters from the traditional approach (subscript “0”) and from Scenario 1 (subscript “1”), respectively. For practical melt processing conditions, the pressure gradient is usually very high, and in such cases, the percentage difference is within 10%. Similar conclusions can be reached from a comparison of the predicted wall shear rate, ${\dot{\gamma }}_{W}=-{\left(dv_{z}\left(r\right)/dr\right)}_{r=R}$, as shown in Figure 9. In obtaining the wall shear rate (${\dot{\gamma }}_{W}$), we used two approaches, one directly solving a nonlinear Equation (by combining Equations (10) and (11)) (shown as lines) and the other from numerical evaluations of ${\dot{\gamma }}_{W}=-{\left(dv_{z}\left(r\right)/dr\right)}_{r=R}$ (shown by symbols) using our numerical solutions of the velocity profile. Both approaches gave the same results.

**Figure 10:**
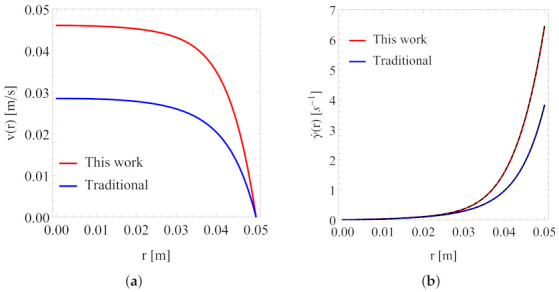
Velocity profile (**a**) and local shear rate (**b**) at pressure gradient = 7.5 MPa/m. Tube radius *R* = 0.05 rm.

**Figure 11:**
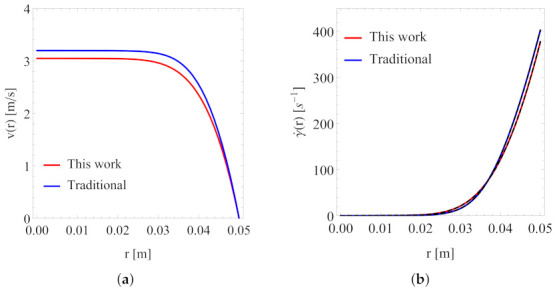
Velocity profile (**a**) and local shear rate (**b**) at pressure gradient = 15 MPa/m. Tube radius *R* = 0.05 rm.

Figure 10 and Figure 11 present a direct comparison of the predicted velocity distribution and local shear rate at low and high pressure gradients, respectively. At a pressure gradient value of 7.5MPa/m, the set of the best-fit viscosity parameters from the traditional approach yields a much lower velocity as compared to that from the set of parameters from Scenario 1. This can be understood from the comparison shown in Figure 7. When the characteristic shear rate (e.g., the wall shear rate) is low, then the difference in the predicted shear viscosity values from the two sets of the best fitting parameters will yield a rather pronounced difference in flow simulations, as demonstrated in Figure 10. However, when the characteristic shear rate is high, then the difference in the predicted shear viscosity values from the two sets of the best fitting parameters in the relevant shear rate range is rather small (within 10%); for this reason, they yield similar results in flow simulations, as demonstrated in Figure 11.

Our results for the local shear rate, as shown in Figure 10b and Figure 11b, were also obtained from two approaches, one from solving a nonlinear Equation (by combining Equations (10) and (11)) directly (shown as solid lines) and the other from numerical evaluations of the velocity gradient (see Equation (12)) from our numerical solutions of the velocity distribution. The latter results are shown as dashed lines in Figure 10b and Figure 11b. Clearly, both approaches gave the same results.

### 3.3. Shear Viscosity as a Function of the Rate of Shear and Temperature

In this subsection, we compared the two sets of best-fitting parameters: one from the traditional approach and the other from the newly proposed iterative approach (Scenario 1), in the thus obtained shear viscosity as a function of the rate of shear and temperature functions. To study non-isothermal flows in practical flow simulations of polymer processing, it is necessary to have $\eta (\dot{\gamma },T)$. To model the temperature dependence, we used the Williams–Landel–Ferry (WLF) [20,22,34] equation, defined as

(15) $$\begin{array}{c} \log_{10}\left(a_{T}^{*}\right)=-\frac{A_{0}\left(T-T_{\mathrm{ref}}\right)}{B_{0}+\left(T-T_{\mathrm{ref}}\right)} \end{array}$$

and:

(16) $$\begin{array}{c} \log_{10}\left(a_{T}\right)=-\frac{A_{1}\left(T-T_{\mathrm{ref}}\right)}{B_{1}+\left(T-T_{\mathrm{ref}}\right)} \end{array}$$

where $A_{0}$, $A_{1}$ are related to the free volume at the reference temperature $T_{\mathrm{ref}}$, and $B_{0}$, $B_{1}$ are related to the thermal expansion’s free-volume coefficient. These estimated parameters for each case in Table 2 are shown in Table 3.

**Figure 12:**
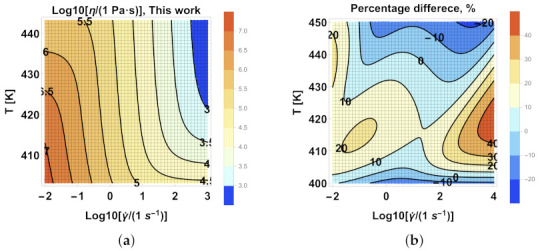
Viscosity vs. shear rate vs. temperature contour plots by the parameters from (**a**) Scenario 1 and (**b**) the percentage difference between Scenario 1 and traditional method.

Figure 12 presents a comparison of the two sets of best-fitting parameters for the $\eta (\dot{\gamma },T)$ function, one from the traditional method and the other from the herein proposed iterative approach (Scenario 1). Here, the percentage difference is defined as

(17) $$\begin{array}{c} \mathrm{PD}=\frac{\log_{10}\left(\frac{\eta_{0}}{\mathrm{Pa}\cdot s}\right)-\log_{10}\left(\frac{\eta_{1}}{\mathrm{Pa}\cdot s}\right)}{\left[\log_{10}\left(\frac{\eta_{0}}{\mathrm{Pa}\cdot s}\right)+\log_{10}\left(\frac{\eta_{1}}{\mathrm{Pa}\cdot s}\right)\right]/2}\times 100\% \end{array}$$

where $\eta_{0}$ is the predicted viscosity by the traditional method and $\eta_{1}$ is the predicted viscosity by Scenario 1. As shown in Figure 12b, the percentage difference, as defined in Equation (diffperc), may exceed 20%, even at large shear rate values.

### 3.4. Nonlinear Regression: Proof of Concept for Scenario 2

**Figure 13:**
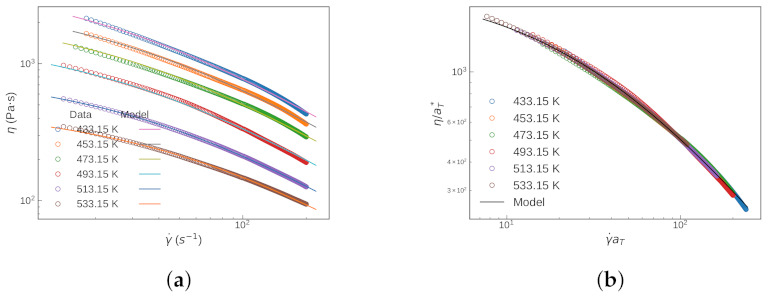
(**a**) Viscosity vs. shear rate at 6 different temperatures; and (**b**) the obtained master curve.

Here, we present a proof-of-concept demonstration for Scenario 2, when the shift factors are not available, based on our in-house measurements (polypropylene sample by SAOS measurements). In Figure 13, we can see the fitted viscosity plots for different temperatures and the corresponding master curve with reference a temperature of T=473.15K. Good fittings were obtained for all temperature values of our measurements Figure 13a, and a good master curve was also obtained—as shown in Figure 13b. Thus, even in cases where prior knowledge of the shifting factors is not available, the newly proposed iterative approach can still lead to a good fit to the experimental data, resulting in both the shifting factors (which can then be used to construct a master curve) and parameters for the non-Newtonian fluid model. Generally speaking, it takes fewer iteration steps to reach a similar goodness-of-fit if we have a good initial guess for the shifting factors, which is the case with Scenario 1 as compared to the case with Scenario 2.

## 4. Conclusions

This work presents a unique iterative algorithm for fitting the rate-of-shear and temperature-dependent viscosity model under the time–temperature superposition (TTS) principle. Proof-of-concept demonstrations were shown using the five-parameter Carreau–Yasuda model and experimental data from small amplitude oscillatory shear measurements. It was shown that the newly proposed iterative algorithm leads to a more accurate representation of the experimental data compared with the traditional approach, judging from all four measures of the goodness of fit that we investigated.

Comparing the two sets of parameters, one from the traditional approach and the other from the newly proposed iterative approach (Scenario 1), and based on the same experimental data in the “isothermal flow in a circular tube” problem, it was found that the two sets of parameters lead to considerable differences in both the velocity distribution and the average flow velocity. The percentage difference between the two predictions can be as large as 10% and above.

Finally, even in cases where prior knowledge of the TTS shifting factors is not available (Scenario 2), the newly proposed iterative approach can still yield a good fitting of the experimental data and the master curve, resulting in both the shifting factors and parameters for the non-Newtonian fluid model. In contrast, the traditional approach requires a two-step procedure (obtaining the shifting factors in the first step and then obtaining the viscosity parameters in the second step) and may not lead to a representation of experimental data as accurate as that from our approach (see Table 2).

In future studies, this newly proposed algorithm may be applied to analyze other types of nonlinear rheological models and it is also worth comparing the performance of those different model parameters which originated from the same experimental data in non-isothermal flow simulations.

## Figures and Tables

**Table 1:** Evolution of the estimated parameters of the Carreau–Yasuda model and TTS shift factors with each iteration (Scenario 1). The values shown for item #1 are referred to as the traditional method in this work.

#	1	2	…	37
$\eta_{\infty }\;\left(\mathrm{Pa}\cdot s\right)$	40,476.13	40,476.32	…	37,190.11
$\eta_{0}\;\left(\mathrm{Pa}\cdot s\right)$	598,346,954.87	598,346,954.87	…	598,346,954.87
*a*	0.7968	0.7981	…	1.2160
*n*	0.1054	0.1059	…	0.1477
λ(s)	4725.29	4726.26	…	6911.72
$a_{T}^{*}$ (403.15 K)	1	1	…	1
$a_{T}$ (403.15 K)	1	1	…	1
$a_{T}^{*}$ (433.15 K)	0.003522	0.003364	…	0.002356
$a_{T}$ (433.15 K)	0.003552	0.003593	…	0.002131
$a_{T}^{*}$ (443.15 K)	0.001023	0.000974	…	0.000753
$a_{T}$ (443.15 K)	0.001089	0.000958	…	0.000636
AIC	−413.93	−446.72	…	−486.74
BIC	−403.54	−436.33	…	−476.35
$adjR^{2}$	0.9994	0.9996	…	0.9998

**Table 2:** Summary of the models with their quality measures.

Model by	AIC	BIC	$R_{\mathrm{adj}}^{2}$	RMSE
Traditional approach using LM	−331.12	−320.74	0.9976	0.0585
Traditional method (with TNC)	−413.93	−403.54	0.9994	0.0318
Global algorithm: Scenario 1 (our method)	−486.74	−476.35	0.9998	0.0148

**Table 3:** Fitted coefficients of WLF for each case.

Model by	$A_{0}$	$A_{1}$	$B_{0}$ (K)	$B_{1}$ (K)
Traditional method	8.7065088	7.97907227	76.4700812	67.72598488
Global algorithm: Scenario 1	7.1865105	7.78847416	52.04696748	57.46612279

## Data Availability

The Python code implementation of the proposed iterative approach is available at https://github.com/medeuamangeldi/Combined-iterative-algorithm-with-Hessian-free-optimization-for-nonlinear-data-fitting-/tree/main (accessed on 28 October 2021). The Wolfram Mathematica notebook file that was used to produce the results shown in Figure 7, Figure 8, Figure 9, Figure 10, Figure 11 and Figure 12 is available in the Supporting Material of this work.

## Supplementary Materials

The following are available at: https://www.mdpi.com/article/10.3390/polym13234185/s1; The Wolfram Mathematica notebook (in PDF format) that we developed to generate the results shown in Figure 7, Figure 8, Figure 9, Figure 10, Figure 11 and Figure 12.

## Abbreviations

The following abbreviations are used in this manuscript:

AIC	Akaike Information Criterion
BIC	Bayesian Information Criterion
CY	Carreau–Yasuda
LM	Levenberg–Marquardt
PD	percentage error between two predicted values
RMSE	root mean squared error
TNC	Truncated–Newton
TTS	time–temperature superposition
