# Supplementary material for: An Iterative Approach for the Parameter Estimation of Shear-Rate and Temperature-Dependent Rheological Models for Polymeric Liquids
Source: Polymers (Basel). 2021 Nov 30;13(23):4185. doi: 10.3390/polym13234185 (PMC8659470; doi:10.3390/polym13234185)
Supplement: Supplementary file 1 [file polymers-13-04185-s001.zip › polymers-1447302-supplementary.pdf]

Supporting Material for “An Iterative Approach for the Parameter Estimation Of Shear-Rate and Temperature-Dependent Viscosity Models for Polymeric Liquids”

Medeu Amangeldi, Yanwei Wang*, Asma Perveen, Dichuan Zhang, and Dongming Wei*

Nazarbayev University, Nur-Sultan 010000, Kazakhstan

Correspondence: yanwei.wang@nu.edu.kz (Y.W.); dongming.wei@nu.edu.kz (D.W.)

1. Viscosity as a function of shear rate and temperature

To clear all definitions of quantities you’ve introduced in a Mathematica session so far, type: ClearAll[“-Global` *”]

```
In[ ]:= ClearAll["Global`*"]
```

$$\log_{10}(a_T^*) = -\frac{A_0(T - T_{ref})}{B_0 + (T - T_{ref})}$$

$$\log_{10}(a_T) = -\frac{A_1(T - T_{ref})}{B_1 + (T - T_{ref})}$$

$$\eta(\dot{\gamma})/a_T^* = \eta_\infty + (\eta_0 - \eta_\infty)\left[1 + (\lambda a_T \dot{\gamma})^a\right]^{\frac{n-1}{a}}$$

#	η_∞	η_0	a	n	λ	a_T^*	a_T	AIC	BIC	$adjR^2$
1	40476.13	598346954.87	0.7968	0.1054	4725.29	1<br>0.003522<br>0.001023	1<br>0.003552<br>0.001089	-413.93	-403.54	0.9994
2	40476.32	598346954.87	0.7981	0.1059	4726.26	1<br>0.003364<br>0.000974	1<br>0.003593<br>0.000958	-446.72	-436.33	0.9996
…	…	…	…	…	…	…	…	…	…	…
37	37190.11	598346954.87	1.2160	0.1477	6911.72	1<br>0.002356<br>0.000753	1<br>0.002131<br>0.000636	-486.74	-476.35	0.9998

Table 4: Fitted coefficients of WLF for each case

Model by	A_0	A_1	B_0	B_1
Traditional method	8.7065088	7.97907227	76.4700812	67.72598488
Global algorithm: *Scenario I*	7.1865105	7.78847416	52.04696748	57.46612279

In[∘]:=
```
Atstar[Temp_, A0_, B0_, Tref_] := 10^(-A0 (Temp - Tref) / (B0 + Temp - Tref));
At[Temp_, A1_, B1_, Tref_] := 10^(-A1 (Temp - Tref) / (B1 + Temp - Tref));
EtaCYTemp[x_, Temp_, EtaInf_, EtaZero_, a_, n_, Lambda_, A0_, B0_, A1_, B1_, Tref_] :=
  (EtaInf + (EtaZero - EtaInf) (1 + (Lambda * (At[Temp, A1, B1, Tref]) * x)^a)^((n-1)/a)) *
   Atstar[Temp, A0, B0, Tref];
```

In[∘]:=
```
figviscosity101 =
  ContourPlot[Log10[EtaCYTemp[10^loggamma, temp, 37190.11, 598346954.87, 1.2160,
     0.1477, 6911.72, 7.1865105, 52.04696748, 7.78847416, 57.46612279, 403.15]],
   {loggamma, -2, 3}, {temp, 403.15, 443.15}, AspectRatio → 1, PlotTheme -> "Business",
   PlotLegends → Automatic, ColorFunction → "LightTemperatureMap",
   ContourLabels → True, PlotPoints → 50, FrameLabel → {"Log10[γ̇/(1 s^-1)]", "T [K]"},
   AxesStyle → Directive[Black, AbsoluteThickness[3]],
   Method → {"DefaultBoundaryStyle" → Automatic,
     "DefaultMeshStyle" → AbsolutePointSize[6], "ScalingFunctions" → None}, Mesh → Full,
   PlotLabel → Style["Log10[η/(1 Pa·s)], This work", Black, FontSize → 16],
   Background → White, ImageSize → 300, AspectRatio → 1,
   BaseStyle → {FontWeight → "Bold", Black, FontSize → 16}]
```

Out[∘]=

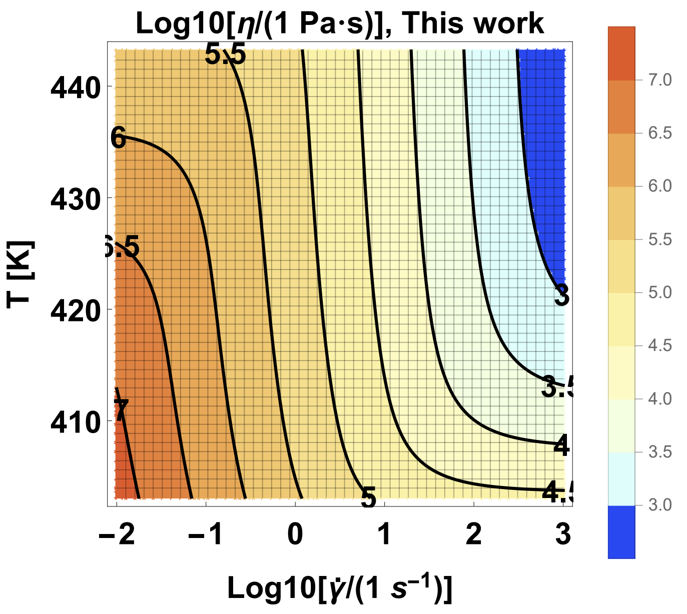

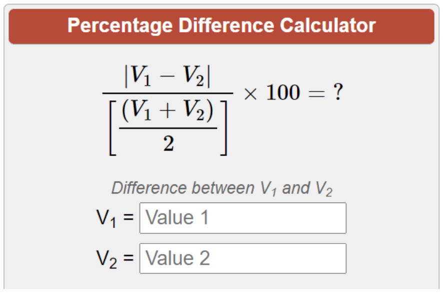

Calculator Use

The Percentage Difference Calculator (% difference calculator) will find the percent difference between two positive numbers greater than 0. Percentage difference is usually calculated when you want to know the difference in percentage between two numbers. For this calculator, the order of the numbers does not matter as we are simply dividing the difference between two numbers by the average of the two numbers. To calculate the difference relatively, from an old number to a new number, you would calculate percentage change.

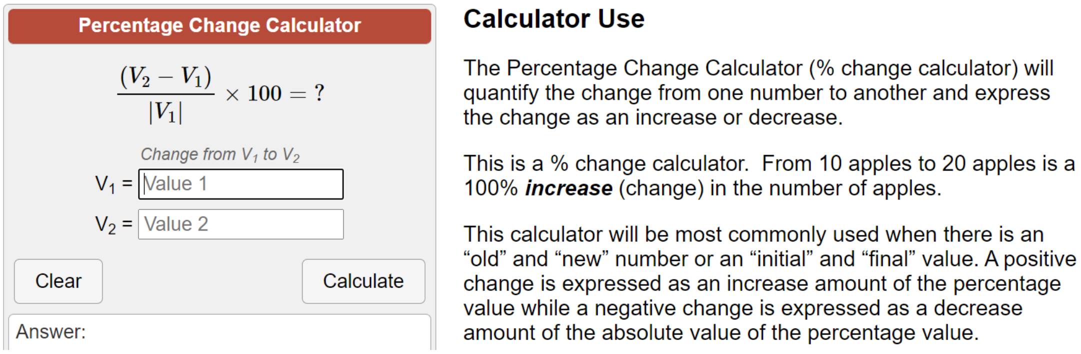

Percentage difference: [η(traditional) - η(this work)]/((η(traditional) + η(this work))/2))

In[∘]:=
```
figviscosity103 =
  ContourPlot[100 * ((EtaCYTemp[10^loggamma, temp, 40476.13, 598346954.87, 0.7968,
         0.1054, 4725.29, 8.7065088, 76.4700812, 7.97907227, 67.72598488, 403.15] -
        EtaCYTemp[10^loggamma, temp, 37190.11, 598346954.87, 1.2160, 0.1477,
         6911.72, 7.1865105, 52.04696748, 7.78847416, 57.46612279, 403.15]) /
      ((EtaCYTemp[10^loggamma, temp, 40476.13, 598346954.87, 0.7968, 0.1054,
           4725.29, 8.7065088, 76.4700812, 7.97907227, 67.72598488, 403.15] +
          EtaCYTemp[10^loggamma, temp, 37190.11, 598346954.87, 1.2160, 0.1477,
           6911.72, 7.1865105, 52.04696748, 7.78847416, 57.46612279, 403.15]) / 2)),
   {loggamma, -2, 4}, {temp, 400, 450}, AspectRatio → 1,
   PlotTheme ->
    "Business", PlotLegends →
    Automatic,
   ColorFunction → "LightTemperatureMap", ContourLabels → True,
   PlotPoints → 50,
   FrameLabel → {"Log10[γ̇/(1 s^-1)]", "T [K]"},
   AxesStyle →
    Directive[RGBColor[0.`, 0.`, 0.`], AbsoluteThickness[3]],
   Method → {"DefaultBoundaryStyle" → Automatic,
     "DefaultMeshStyle" → AbsolutePointSize[6], "ScalingFunctions" → None},
   Mesh → Full, PlotLabel → Style["Percentage differece, %", Black, FontSize → 16],
   Background → White,
   ImageSize → 300,
   AspectRatio → 1,
   BaseStyle → {FontWeight → "Bold", Black, FontSize → 16}]
```

Out[∘]=

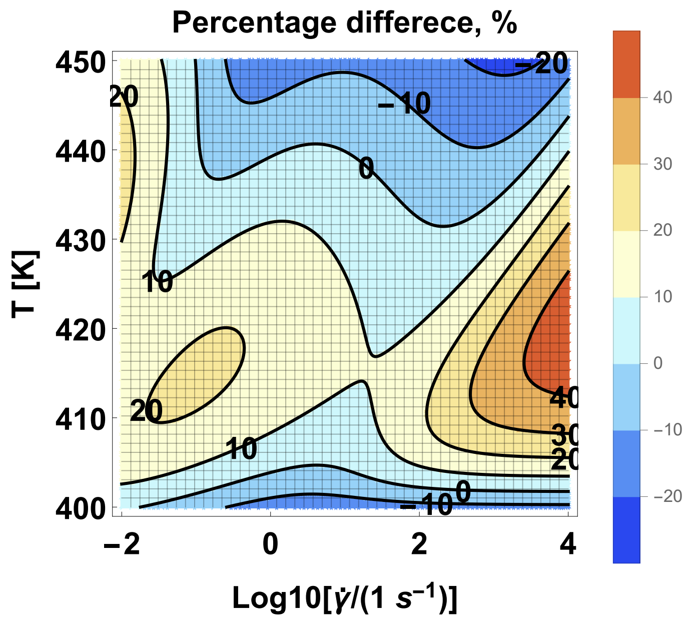

In[∘]:=
```
Export["ShearRateTemperatureViscosity.pdf",
  figviscosity101, "AllowRasterization" → True, ImageResolution → 600];
Export["ShearRateTemperatureViscosityPD.pdf", figviscosity103,
  "AllowRasterization" → True, ImageResolution → 600];
```

2. Comparison on the predicted Shear Viscosity, isothermal case (“403.15 K”,“433.15 K”,“443.15 K”)

To clear all definitions of quantities you’ve introduced in a Mathematica session so far, type: ClearAll[“-Global`*”]

In[∘]:=
```
ClearAll["Global`*"]
```

In[∘]:=
```
EtaCY[x_, EtaInf_, EtaZero_, a_, n_, Lambda_, atstar_, at_] :=
  (EtaInf + (EtaZero - EtaInf) (1 + (Lambda * at * x)^a)^((n-1)/a)) * atstar;
(** Define the C-Y model viscosity function **)
```

In[∘]:=
```
figviscosity201 =
 LogLogPlot[{EtaCY[x, 37190.11, 598346954.87, 1.2160, 0.1477, 6911.72, 1, 1],
   EtaCY[x, 40476.13, 598346954.87, 0.7968, 0.1054, 4725.29, 1, 1],
   EtaCY[x, 37190.11, 598346954.87, 1.2160, 0.1477, 6911.72, 0.002356, 0.002131],
   EtaCY[x, 40476.13, 598346954.87, 0.7968, 0.1054, 4725.29, 0.003522, 0.003552],
   EtaCY[x, 37190.11, 598346954.87, 1.2160, 0.1477, 6911.72, 0.000753, 0.000636],
   EtaCY[x, 40476.13, 598346954.87, 0.7968, 0.1054, 4725.29, 0.001023, 0.001089]},
  {x, 10^-2, 10^4}, Frame → True, AxesOrigin → {0, 0},
  PlotStyle → {{Black, Thick}, {Black, Dashed, Thick}, {Blue, Thick},
    {Blue, Dashed, Thick}, {Red, Thick}, {Red, Dashed, Thick}},
  GridLines → None, FrameLabel → {"γ̇ [s^-1]", "η [Pa·s]"},
  LabelStyle → Directive[FontFamily → "Times", FontSize → 16, Black],
  PlotLegends → Placed[{"This work", "Traditional"}, {0.70, 0.85}],
  Background → White, ImageSize → 300, AspectRatio → 1,
  BaseStyle → {FontWeight → "Bold", Black, FontSize → 16}]
```

Out[∘]=

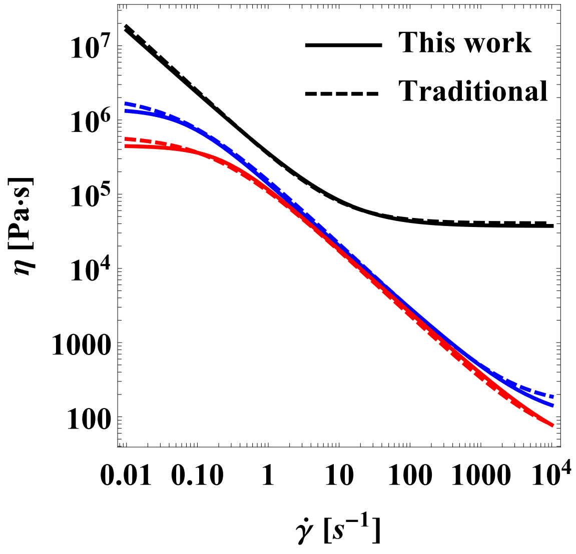

In[∘]:=
```
pdt1[x_] := 100 * (EtaCY[x, 40476.13, 598346954.87, 0.7968, 0.1054, 4725.29, 1, 1] -
      EtaCY[x, 37190.11, 598346954.87, 1.2160, 0.1477, 6911.72, 1, 1]) /
    ((EtaCY[x, 40476.13, 598346954.87, 0.7968, 0.1054, 4725.29, 1, 1] +
        EtaCY[x, 37190.11, 598346954.87, 1.2160, 0.1477, 6911.72, 1, 1]) / 2);
pdt2[x_] := 100 * (EtaCY[x, 40476.13, 598346954.87, 0.7968,
        0.1054, 4725.29, 0.003522, 0.003552] -
      EtaCY[x, 37190.11, 598346954.87, 1.2160, 0.1477, 6911.72, 0.002356, 0.002131]) /
    ((EtaCY[x, 40476.13, 598346954.87, 0.7968, 0.1054, 4725.29, 0.003522, 0.003552] +
        EtaCY[x, 37190.11, 598346954.87, 1.2160,
         0.1477, 6911.72, 0.002356, 0.002131]) / 2);
pdt3[x_] := 100 * (EtaCY[x, 40476.13, 598346954.87, 0.7968, 0.1054,
        4725.29, 0.001023, 0.001089] -
      EtaCY[x, 37190.11, 598346954.87, 1.2160, 0.1477, 6911.72, 0.000753, 0.000636]) /
    ((EtaCY[x, 40476.13, 598346954.87, 0.7968, 0.1054, 4725.29, 0.001023, 0.001089] +
        EtaCY[x, 37190.11, 598346954.87, 1.2160,
         0.1477, 6911.72, 0.000753, 0.000636]) / 2);
```

In[∘]:=
```
figviscosity202 = LogLinearPlot[{pdt1[x], pdt2[x], pdt3[x]},
  {x, 10^-2, 10^4}, Frame → True, AxesOrigin → {0, 0}, PlotRange → {-20, 30},
  PlotStyle → {{Black, Thick}, {Blue, Thick}, {Red, Thick}},
  GridLines → None, FrameLabel → {"γ̇ [s^-1]", "Percentage difference, %"},
  LabelStyle → Directive[FontFamily → "Times", FontSize → 16, Black],
  PlotLegends → Placed[{"403.15 K", "433.15 K", "443.15 K"}, {0.70, 0.80}],
  Background → White, ImageSize → 300, AspectRatio → 1]
```

Out[∘]=

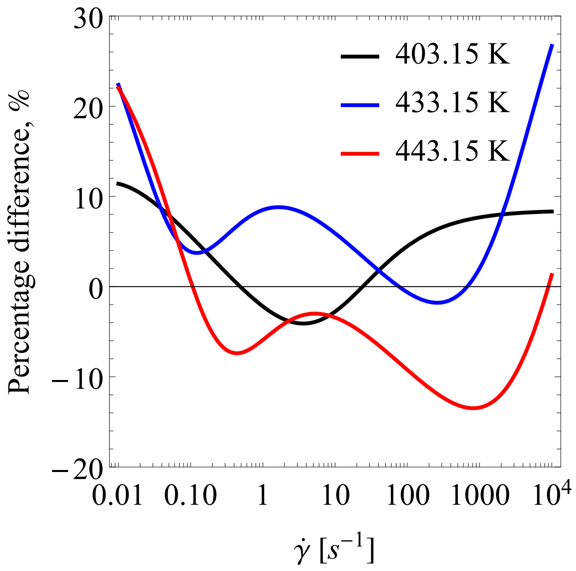

In[∘]:=
```
Export["figviscositycomparison.pdf", figviscosity201,
  "AllowRasterization" → True, ImageResolution → 600];
Export["figviscosityPD.pdf", figviscosity202,
  "AllowRasterization" → True, ImageResolution → 600];
```

In[∘]:=
```
SystemOpen[DirectoryName[AbsoluteFileName["figviscositycomparison.pdf"]]]
```

3. Average Flow Velocity and Shear Rate at the Wall, isothermal case (“433.15 K”)

3.1 Average Flow Velocity

To clear all definitions of quantities you’ve introduced in a Mathematica session so far, type: ClearAll[“-Global`*”]

In[ ]:=
```
ClearAll["Global`*"]
```

In[ ]:=
```
EtaCY[x_, EtaInf_, EtaZero_, a_, n_, Lambda_, atstar_, at_] :=
  (EtaInf + (EtaZero - EtaInf) (1 + (Lambda * at * x)^a)^((n-1)/a)) * atstar;
(** Define the C-Y model viscosity function **)
```

In[ ]:=
```
PipeRadius = 0.05;(**Pipe radius m**)
CrossSection = Pi * PipeRadius^2;
M = 191;
Do[
  PGradient[i] = 1 + (i - 1) * 0.1 (**MPa/m**);
  (**ode01=(-D[vz[r],r])*EtaCY[Abs[D[vz[r],r]],37079.27, 449711022.23,
      1.241, 0.15, 5064.697,0.00311,0.00293]==0.5*PGradient[i]*10^6*r;
  ysol01=NDSolveValue[{ode01,vz[PipeRadius ]==0},vz,{r,0,PipeRadius},
    Method->{"EquationSimplification"->"Residual"}];
  WallShearRate01[i]=(-D[ysol01[r],r]/.r→PipeRadius);
  AvgVel01[i]=NIntegrate[ysol01[r]*2*Pi*r,{r,0,PipeRadius}]/CrossSection ;**)
  ode02 = (-D[vz[r], r]) * EtaCY[Abs[D[vz[r], r]], 37190.11, 598346954.87,
      1.2160, 0.1477, 6911.72, 0.002356, 0.002131] == 0.5 * PGradient[i] * 10^6 * r;
  ysol02 = NDSolveValue[{ode02, vz[PipeRadius ] == 0}, vz, {r, 0, PipeRadius},
    Method -> {"EquationSimplification" -> "Residual"}];
  WallShearRate02[i] = (-D[ysol02[r], r] /. r → PipeRadius);
  AvgVel02[i] = NIntegrate[ysol02[r] * 2 * Pi * r, {r, 0, PipeRadius}] / CrossSection ;
  ode03 = (-D[vz[r], r]) * EtaCY[Abs[D[vz[r], r]], 40476.13, 598346954.87,
      0.7968, 0.1054, 4725.29, 0.003522, 0.003552] == 0.5 * PGradient[i] * 10^6 * r;
  ysol03 = NDSolveValue[{ode03, vz[PipeRadius ] == 0}, vz, {r, 0, PipeRadius},
    Method -> {"EquationSimplification" -> "Residual"}];
  WallShearRate03[i] = (-D[ysol03[r], r] /. r → PipeRadius);
  AvgVel03[i] = NIntegrate[ysol03[r] * 2 * Pi * r, {r, 0, PipeRadius}] / CrossSection ;
  , {i, 1, M}];
```

In[◦]:=
```
Figure6a = ListPlot[{Table[{PGradient[i], AvgVel02[i]}, {i, 1, M}],
    Table[{PGradient[i], AvgVel03[i]}, {i, 1, M}]},
  PlotMarkers → {{○, 6}, {□, 6}}, PlotStyle → { Red, Blue}, GridLines → None,
  PlotRange → All, GridLinesStyle → Directive[Dashed],
  Frame → True, FrameLabel → {"ΔP/L  [MPa/m]", "v_avg   [m/s]"},
  LabelStyle → Directive[FontFamily → "Times", FontSize → 18, Black],
  Background → White, ImageSize → 300, AspectRatio → 1,
  PlotLegends → Placed[{"This work", "Traditional"}, {0.25, 0.75}]]
```

Out[◦]=

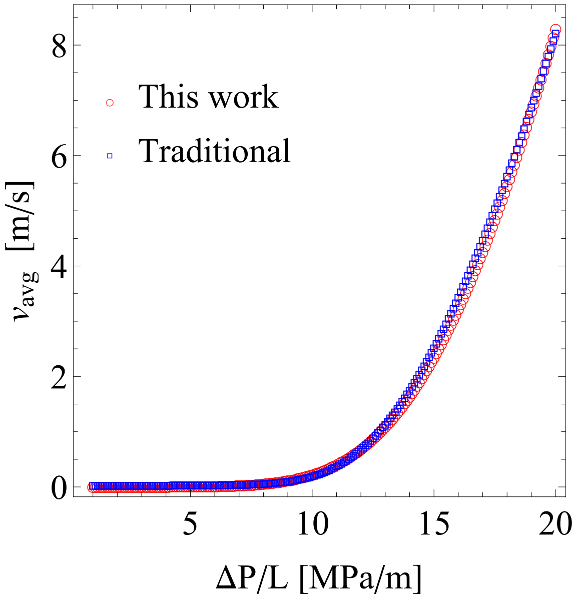

In[◦]:=
```
PercentageErrorVel =
  Table[{PGradient[i], 100 * (AvgVel03[i] - AvgVel02[i]) / ((AvgVel02[i] + AvgVel03[i]) / 2)},
   {i, 1, M}]; (** Percentage difference **)
```

In[ ]:=
```
Figure6b = ListPlot[PercentageErrorVel, PlotMarkers → {{□, 6}}, PlotStyle → {Black},
  GridLines → None, PlotRange → {-60, 40}, GridLinesStyle → Directive[Dashed],
  Frame → True, FrameLabel → {"ΔP/L [MPa/m]", "Percentage difference, %"},
  LabelStyle → Directive[FontFamily → "Times", FontSize → 16, Black],
  Background → White, ImageSize → 300, AspectRatio → 1,
  PlotLegends → Placed[{"in average velocity"}, {0.60, 0.87}]]
```

Out[ ]=

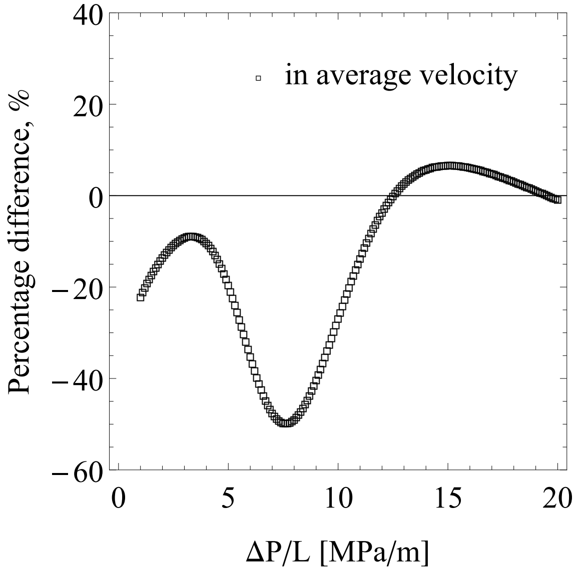

In[ ]:=
```
Export["Vavg.pdf", Figure6a, "AllowRasterization" → True, ImageResolution → 600];
Export["VavgPD.pdf", Figure6b, "AllowRasterization" → True, ImageResolution → 600];
```

In[ ]:=
```
SystemOpen[DirectoryName[AbsoluteFileName["Vavg.pdf"]]]
```

3.2 Shear Rate at the Wall

In[•]:=

```
dM = 2;
oldFigure7a = ListPlot[{Table[{PGradient[i], Log10[WallShearRate02[i]]}, {i, 1, M, dM}],
    Table[{PGradient[i], Log10[WallShearRate03[i]]}, {i, 1, M, dM}]}, PlotMarkers →
    {{○, 6}, {□, 6}}, PlotStyle → { {Red, Thick}, {Blue, Thick}}, PlotRange → {-2, 3},
   Frame → True, FrameLabel → {"ΔP/L  [MPa/m]", "Log10[γ̇W/(1 s⁻¹)]"}, LabelStyle →
    Directive[FontFamily → "Times", FontSize → 16, Black], Background → White, ImageSize →
    300, AspectRatio → 1, PlotLegends → Placed[{"This work", "Traditional"}, {0.70, 0.15}]]
```

Out[•]=

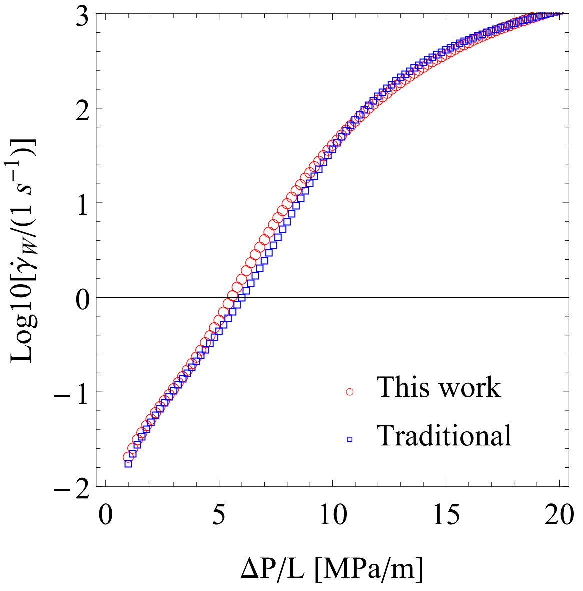

In[•]:=
```
Figure7acheck =
 ContourPlot[{0.5 * x * 10^6 * PipeRadius == EtaCY[10^y, 37190.11, 598346954.87, 1.2160,
      0.1477, 6911.72, 0.002356, 0.002131] * 10^y, 0.5 * x * 10^6 * PipeRadius ==
    EtaCY[10^y, 40476.13, 598346954.87, 0.7968, 0.1054, 4725.29, 0.003522, 0.003552] * 10^y},
  {x, 1, 20}, {y, -2, 3}, ContourStyle → { {Red, Thick}, {Blue, Thick}},
  Contours → 100, WorkingPrecision -> MachinePrecision, PlotPoints → 200,
  Frame → True, FrameLabel → {"ΔP/L  [MPa/m]", "Log10[γ̇W/(1 s^-1)]"},
  LabelStyle → Directive[FontFamily → "Times", FontSize → 16, Black],
  Background → White, ImageSize → 300, AspectRatio → 1 ]
 (** y = log10(shear rate), x = Pressure gradient **)
```

Out[•]=

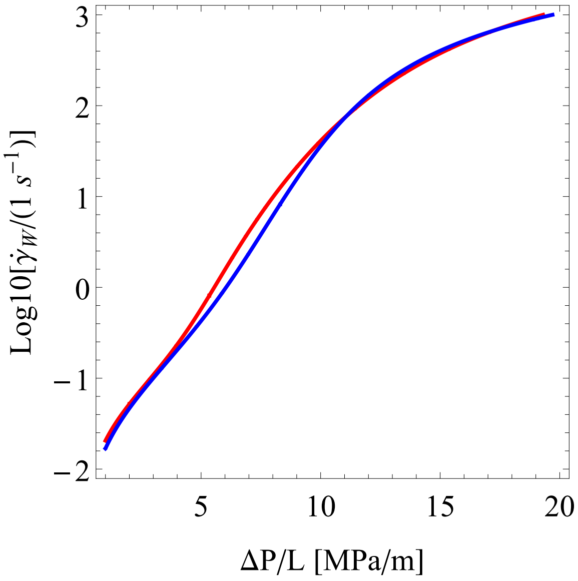

In[•]:=
```
Figure7a = Show[oldFigure7a, Figure7acheck]
```

Out[•]=

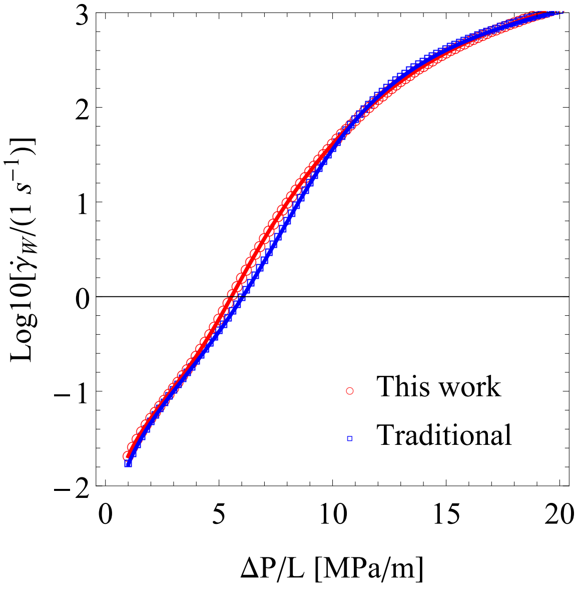

In[]:=
```
PDGammaWall = Table[{PGradient[i], 100 * (WallShearRate03[i] - WallShearRate02[i]) /
      ((WallShearRate03[i] + WallShearRate02[i]) / 2)}, {i, 1, M}];
Figure7b = ListPlot[{PDGammaWall}, PlotMarkers → {{□, 6}}, PlotStyle → {Black},
  GridLines → None, PlotRange → {-60, 40}, GridLinesStyle → Directive[Dashed],
  Frame → True, FrameLabel → {"ΔP/L [MPa/m]", "Percentage difference, %"},
  LabelStyle → Directive[FontFamily → "Times", FontSize → 16, Black],
  Background → White, ImageSize → 300, AspectRatio → 1,
  PlotLegends → Placed[{"in wall shear rate"}, {0.60, 0.87}]]
```

Out[]=

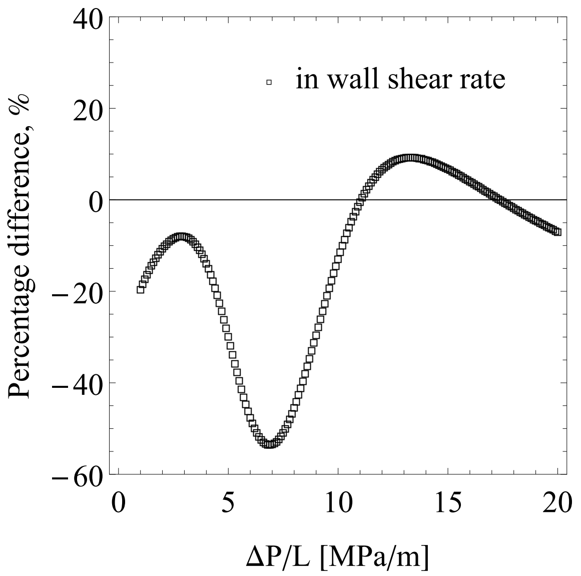


```
Export["WallShearRate.pdf", Figure7a, "AllowRasterization" → True, ImageResolution → 600];
Export["WallShearRatePD.pdf", Figure7b, "AllowRasterization" → True, ImageResolution → 600];
```

ShearRateTemperatureViscosity

3.1 Velocity and Shear Rate Distribution at Low Pressure Gradient

In[]:=
```
ClearAll["Global`*"]
```

In[]:=
```
EtaCY[x_, EtaInf_, EtaZero_, a_, n_, Lambda_, atstar_, at_] :=
  (EtaInf + (EtaZero - EtaInf) (1 + (Lambda * at * x)^a)^((n-1)/a)) * atstar;
(** Define the C-Y model viscosity function **)
```

In[]:=
```
PGradient = 7.5 * 10^6 (**=(P0-PL)/L = Pressure gradient, [
Pa/m]**); R = 0.05 (**Pipe radius [m]**);
```

In[•]:=
```
(**ode1=(-D[vz[r],r])*EtaCY[Abs[D[vz[r],r]],37079.27,
    449711022.23,1.241,0.15,5064.697,0.00311,0.00293]==0.5*PGradient*r;
ysol1=NDSolveValue[{ode1,vz[R]==0},vz,{r,0,R},
  Method->{"EquationSimplification"->"Residual"}];**)

ode2 = (-D[vz[r], r]) * EtaCY[Abs[D[vz[r], r]], 37190.11, 598346954.87,
     1.2160, 0.1477, 6911.72, 0.002356, 0.002131] == 0.5 * PGradient * r;
ysol2 = NDSolveValue[{ode2, vz[R] == 0}, vz, {r, 0, R},
   Method -> {"EquationSimplification" -> "Residual"}];

ode3 = (-D[vz[r], r]) * EtaCY[Abs[D[vz[r], r]], 40476.13, 598346954.87,
     0.7968, 0.1054, 4725.29, 0.003522, 0.003552] == 0.5 * PGradient * r;
ysol3 = NDSolveValue[{ode3, vz[R] == 0}, vz, {r, 0, R},
   Method -> {"EquationSimplification" -> "Residual"}];
```

In[•]:=
```
Figure8a = Plot[{ysol2[r], ysol3[r]}, {r, 0, R}, FrameLabel → {"r  [m]", "v(r)  [m/s]"},
  PlotStyle → {{Red, Thick}, {Blue, Thick}}, GridLines → None, PlotRange → {0, 0.05},
  Frame → True, LabelStyle → Directive[FontFamily → "Times", FontSize → 16, Black],
  Background → White, ImageSize → 300, AspectRatio → 1,
  PlotLegends → Placed[{"This work", "Traditional"}, {0.25, 0.2}]]
```

Out[•]=

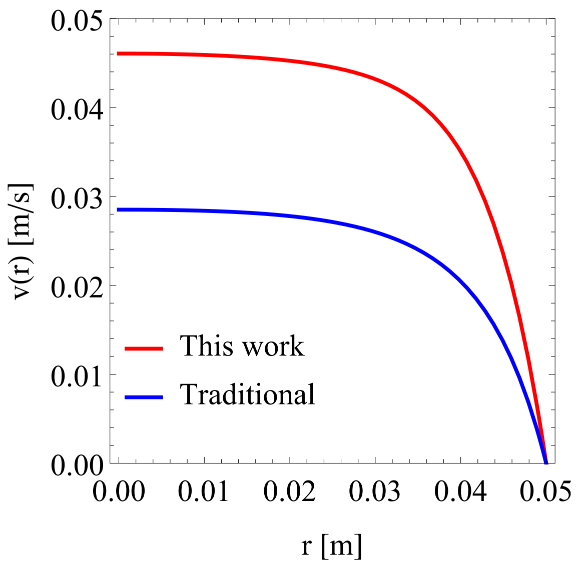

In[•]:=
```
Figure8b1 = Plot[{(-D[ysol2[r], r] /. r → s), (-D[ysol3[r], r] /. r → s)}, {s, 0, R},
  FrameLabel → {"r  [m]", "γ̇(r)  [s^-1]"}, PlotStyle → {{Red, Thick}, {Blue, Thick}},
  GridLines → None, PlotRange → {0, 7}, Frame → True,
  LabelStyle → Directive[FontFamily → "Times", FontSize → 16, Black],
  Background → White, ImageSize → 300, AspectRatio → 1,
  PlotLegends → Placed[{"This work", "Traditional"}, {0.25, 0.80}]]
```

Out[•]=

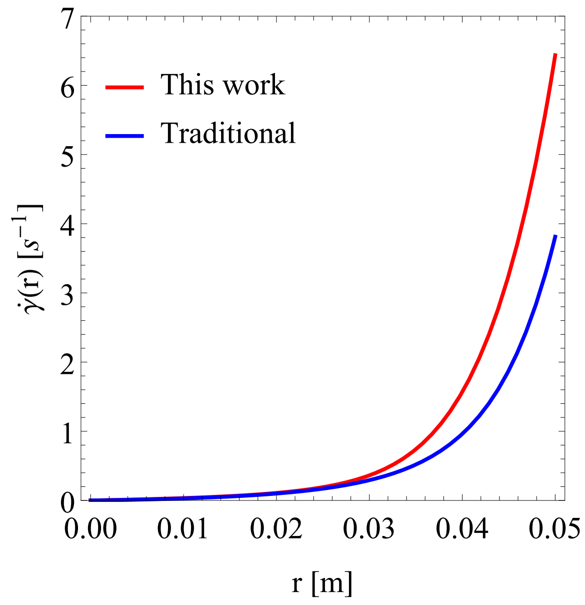


```
(** Contours→50,WorkingPrecision->MachinePrecision,PlotPoints→200**)
```

In[∘]:=
```
Figure8b2 =
 ContourPlot[{0.5 * PGradient * r == EtaCY[s, 37190.11, 598346954.87, 1.2160, 0.1477,
       6911.72, 0.002356, 0.002131] * s, 0.5 * PGradient * r ==
     EtaCY[s, 40476.13, 598346954.87, 0.7968, 0.1054, 4725.29, 0.003522, 0.003552] * s},
   {r, 0, 0.05}, {s, 0, 7}, ContourStyle → {{Black, Dashed}, {Black, Dashed}},
   Contours → 100, WorkingPrecision -> MachinePrecision,
   PlotPoints → 200, GridLines → None, Frame → True,
   LabelStyle → Directive[FontFamily → "Times", FontSize → 16, Black],
   Background → White, ImageSize → 300, AspectRatio → 1]
 (** s = gamma dot, shear rate **)
```

Out[∘]=

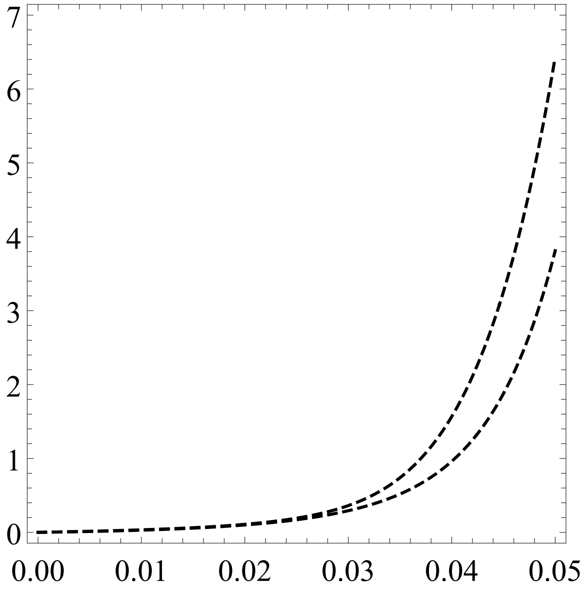

In[∘]:=
```
Figure8b = Show[Figure8b1, Figure8b2]
```

Out[∘]=

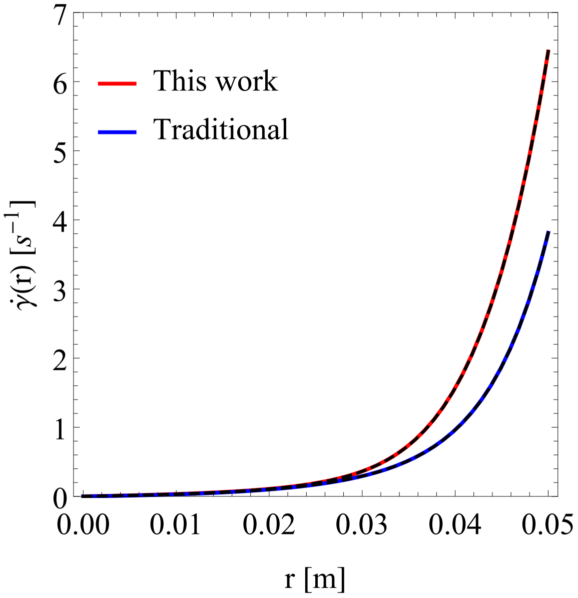

```
In[]:= Export["VelocityLowPressure.pdf", Figure8a,
         "AllowRasterization" → True, ImageResolution → 600];
       Export["ShearRateLowPressure.pdf", Figure8b,
         "AllowRasterization" → True, ImageResolution → 600];
```

3.2 Velocity and Shear Rate Distribution at High Pressure Gradient

```
In[]:= ClearAll["Global`*"]
```

```
In[]:= EtaCY[x_, EtaInf_, EtaZero_, a_, n_, Lambda_, atstar_, at_] :=
         (EtaInf + (EtaZero - EtaInf) (1 + (Lambda * at * x)^a)^((n-1)/a)) * atstar;
       (** Define the C-Y model viscosity function **)
```

```
In[]:= PGradient = 15 * 10^6 (**= (P0-PL)/L = Pressure gradient, [
       Pa/m]**); R = 0.05 (**Pipe radius [m]**);
```

```
In[]:= (**ode1=(-D[vz[r],r])*EtaCY[Abs[D[vz[r],r]],37079.27,
          449711022.23,1.241,0.15,5064.697,0.00311,0.00293]==0.5*PGradient*r;
       ysol1=NDSolveValue[{ode1,vz[R]==0},vz,{r,0,R},
         Method->{"EquationSimplification"->"Residual"}];**)

       ode2 = (-D[vz[r], r]) * EtaCY[Abs[D[vz[r], r]], 37190.11, 598346954.87,
            1.2160, 0.1477, 6911.72, 0.002356, 0.002131] == 0.5 * PGradient * r;
       ysol2 = NDSolveValue[{ode2, vz[R] == 0}, vz, {r, 0, R},
          Method -> {"EquationSimplification" -> "Residual"}];

       ode3 = (-D[vz[r], r]) * EtaCY[Abs[D[vz[r], r]], 40476.13, 598346954.87,
            0.7968, 0.1054, 4725.29, 0.003522, 0.003552] == 0.5 * PGradient * r;
       ysol3 = NDSolveValue[{ode3, vz[R] == 0}, vz, {r, 0, R},
          Method -> {"EquationSimplification" -> "Residual"}];
```

In[•]:=
```
Figure9a = Plot[{ysol2[r], ysol3[r]}, {r, 0, R}, FrameLabel → {"r  [m]", "v(r)  [m/s]"},
  PlotStyle → {{Red, Thick}, {Blue, Thick}}, GridLines → None, PlotRange → {0, 4},
  Frame → True, LabelStyle → Directive[FontFamily → "Times", FontSize → 16, Black],
  Background → White, ImageSize → 300, AspectRatio → 1,
  PlotLegends → Placed[{"This work", "Traditional"}, {0.25, 0.2}]]
```

Out[•]=

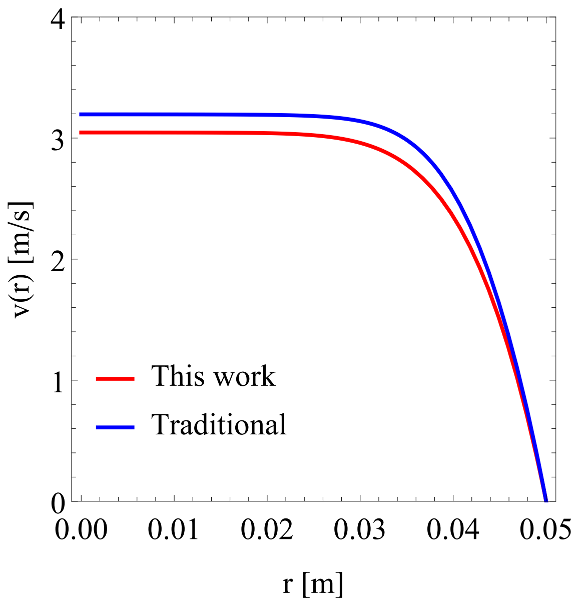

In[•]:=
```
Figure9b1 = Plot[{(-D[ysol2[r], r] /. r → s), (-D[ysol3[r], r] /. r → s)}, {s, 0, R},
  FrameLabel → {"r  [m]", "γ̇(r)  [s^-1]"}, PlotStyle → {{Red, Thick}, {Blue, Thick}},
  GridLines → None, PlotRange → {0, 450}, Frame → True,
  LabelStyle → Directive[FontFamily → "Times", FontSize → 16, Black],
  Background → White, ImageSize → 300, AspectRatio → 1,
  PlotLegends → Placed[{"This work", "Traditional"}, {0.25, 0.80}]]
```

Out[•]=

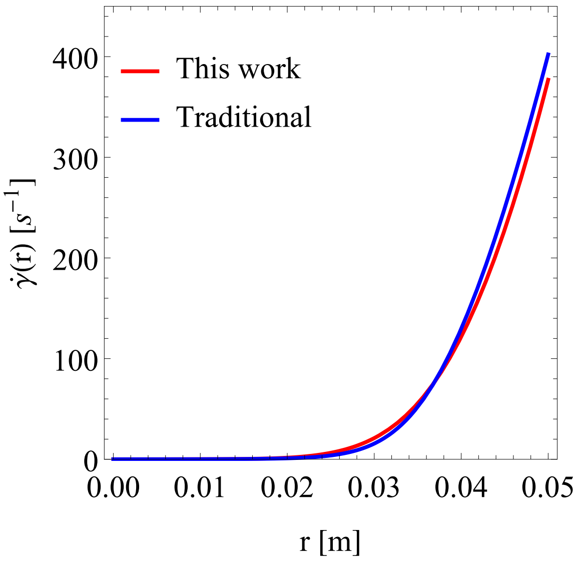

In[•]:=
```
(** Contours→50,WorkingPrecision->MachinePrecision,PlotPoints→200**)
```

In[∘]:=
```
Figure9b2 =
  ContourPlot[{0.5 * PGradient * r == EtaCY[s, 37 190.11, 598 346 954.87, 1.2160, 0.1477,
        6911.72, 0.002356, 0.002131] * s, 0.5 * PGradient * r ==
      EtaCY[s, 40 476.13, 598 346 954.87, 0.7968, 0.1054, 4725.29, 0.003522, 0.003552] * s},
   {r, 0, 0.05}, {s, 0, 450}, ContourStyle → {{Black, Dashed}, {Black, Dashed}},
   Contours → 100, WorkingPrecision -> MachinePrecision,
   PlotPoints → 200, GridLines → None, Frame → True,
   LabelStyle → Directive[FontFamily → "Times", FontSize → 16, Black],
   Background → White, ImageSize → 300, AspectRatio → 1]
  (** s = gamma dot, shear rate **)
```

Out[∘]=

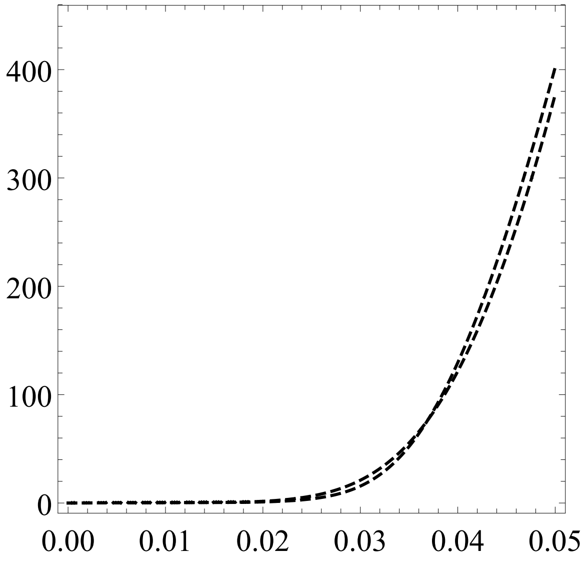

In[∘]:=
```
Figure9b = Show[Figure9b1, Figure9b2]
```

Out[∘]=

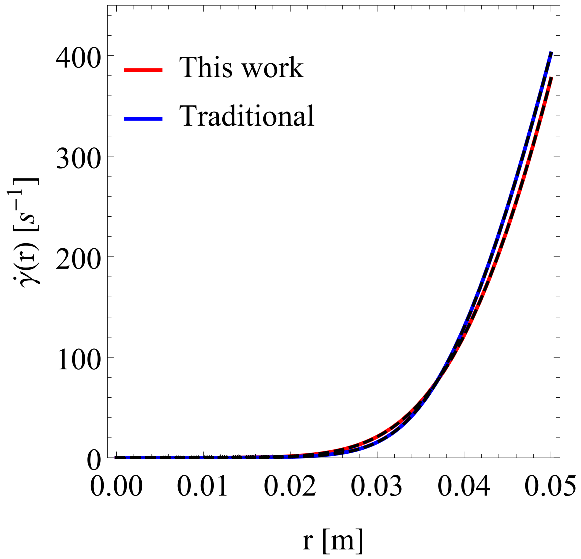

In[∘]:=
```
Export["VelocityHighPressure.pdf", Figure9a,
   "AllowRasterization" → True, ImageResolution → 600] ;
Export["ShearRateHighPressure.pdf", Figure9b,
   "AllowRasterization" → True, ImageResolution → 600] ;
```

In[ ]:=
```
SystemOpen[DirectoryName[AbsoluteFileName["VelocityHighPressure.pdf"]]]
```

This is the END. (Last update by Yanwei Wang, November 1, 2021)
